# The Poverty of Dentists

**Published:** 1858-03

**Authors:** 


### THE POVERTY OF DENTISTS.

From a general observation it will be found that the great mass of dentists are of very limited means, and this, too, in spite of its practice being the most lucrative of all the professions. True we do not get a Russian Diplomatic case, but we occasionlly obtain the patronage of an Emperor and receive a mimic crown set with precious stones, as well as other tokens of the gratitude of the great rulers of earth.

These are like the tears of angels, soothing to our misfortunes, and like the stars of heaven, only for the poor to gaze at, yet we are satisfied from long observation that the absence of the luxuries of wealth, is mostly a self-imposed want, as begotten by great imprudence, and as the result of blind and headlong folly.

Young men when they have fulfilled their studentship, perhaps under great privations, commence practice mostly with but moderate advantages, as to outfit, often with little or nothing. However, they soon manage to obtain a small practice, at first difficult to procure, but little by little it comes, and they feel that they have at last learned the great secret of making money. Beginning to feel the want of various supplies they obtain them on credit, thinking that they can soon pay the debt, and so they would, but this same confidence induces them to buy many other things of all sorts until they soon acquire the habit of wanting this and that comparatively useless matter, much faster than their income justifies. They seldom reflect upon any worthy object in their future, seldom hold the acquisition of professional attainment as the highest point in time to come, but lunge one day into another regardless of anything but idle and personal gratifications.

Thoughtlessly they exist one year after another, until a proper maturity of age finds them, perhaps, with no reputation, resorting to doubtful practices and worse customs to obtain what business they enjoy, less capable, less honorable, and more unworthy of patronage than when they commenced, and with thousands of miserable operations to reproach them, pronouncing death to the little remaining hopes of the future.

It now becomes a struggle to live, for they have been sedulously earning the weightest burden to carry—a bad reputation, nor is one man in a thousand as capable to relieve himself as the donkey is the firmly girted pannier. Perhaps we are telling our own experience, so much the better, it will be more absolute, and at any rate we certainly can speak as one who has gone before many on the road to proffer experience. We certainly do speak as one who has suffered no little from prodigality, and one who has seen numberless cases of ruin and defeat, in persons of more than ordinary natural ability, who have fallen to this early weakness of a want of strict economy.

It would be almost folly for one already steeped in ruin and dismay to read this subject, as he is too far gone for recovery, but we offer these suggestions to the younger men, who are coming forward in such numbers, to have a care over their time and money, from the first moment of entering practice.

Our profession is becoming filled with men of educational ability, men who unite theory and practice, men who are well versed in general science and the important laws of mechanics. We have men who are peculiarly practical in all their acquirements, and who study the useful applicability of all sciences to our art. You must devote all your leisure time to these purposes, in order to keep pace with such, and to do this you must economise your means. I know no better plan than saving at least *half* of your receipts, let your wants be what they may.

Such a course will find you in only six years, rich in pocket, rich in professional knowledge, but best of all, rich in friends, for this world is so constituted as to love those best who have the most money. Think of this source of power and of the immense usefulness you can be to a community and to your calling.

Acquire knowledge by all means in your control, but money, honestly first, if possible, have it at all costs, but the sacrifice of moral principle, because with it you can acquire all things that are useful, and make your attainments most servicable to the wants of man. With it you can do all things that nature or your ambition prompts you to, imbibe knowledge, obtain power, command business, defeat your enemies, laugh at misfortune, or only admire the beautiful at your ease.

The idea that money is the "root of all evil," is proven every day a fallacy, on the contrary, every sane man confesses it to be the *great necessity* of existence. The abuse of it is the evil, as the abuse is the defeat of many wise domestic and political institutions, therefore run not into the error of misusing the money you toil for, but guard it prudently by never *lending*, simply give when you have to much, or when a fellow being is suffering from its want, and by saving always a generous portion of your earnings.

I know of nothing successful without it; a talented man who is *poor* is as much under par in our professions, as wild cat paper in Wall street, a poor lover, a poor husband, a *poor dentist* is out of date, thrown by for richer ones by all.

This is a verity, one that always has been, and always will be, be poor, and however honest, however industrous, however talented and learned I care not, you will be little better than nothing until you can show a respectable balance in bank. Let no one argue or ridicule you out of this impression, but act upon it and "put money in thy pocket." Money makes great statesmen, great ministers, great physicians *great dentists*, as well as little men great generally, and if you, reader, would be well thought of, and well spoken of by all, get rich. The purest sentiments of your breast, shall be thrilled by gentler, sweeter lips, through its magic powers. Your avocation will be tenfold more pleasant and courted, even the church made more congenial and warm.

Money is the mysterious ægis of every quality aiming at success in every direction, and it is *impossible* to overrate its importance.

Our profession only needs its influence, to equal in standing all others; our schools need it to prosper, our art requires it to reach a higher perfection than all other arts, because we have not only the domain of *beauty* in our charge, but we have immediate usefulness also, as we should always restore the lost functions entire of a part, not make a passable substitute, or a faulty imitation. The surgeon *may* remove what he never restores, we remove that which we *always can* restore.

A medical patient limps through the world in a crippled condition of body or limb, ours trip along happy and perfect in the restoration of youths highest adornment, and most essential possession, remembering always, reader, that they love and protect best that *which has cost them the most.* A.

